# Propranolol induces a favourable shift of anti-tumor immunity in a murine spontaneous model of melanoma

**DOI:** 10.18632/oncotarget.12833

**Published:** 2016-10-24

**Authors:** Ludovic Jean Wrobel, Lloyd Bod, Renée Lengagne, Masashi Kato, Armelle Prévost-Blondel, Frédérique-Anne Le Gal

**Affiliations:** ^1^ Hôpitaux Universitaires de Genève, Service de Dermatologie, Genève, Switzerland; ^2^ Inserm, U1016, Institut Cochin, Paris, France; ^3^ CNRS, UMR8104, Paris, France; ^4^ Université Paris Descartes, Paris, France; ^5^ Department of Occupational and Environmental Health, Nagoya University Graduate School of Medicine, Aichi, Japan

**Keywords:** melanoma, beta-blocker, norepinephrine, anti-tumor immunity, propranolol

## Abstract

In a previous study on a xenograft model of melanoma, we showed that the beta-adrenergic receptor antagonist propranolol inhibits melanoma development by modulating angiogenesis, proliferation and cell survival. Stress hormones can influence tumor development in different ways and norepinephrine was shown to downregulate antitumor immune responses by favoring the accumulation of immunosuppressive cells, impairing the function of lymphocytes. We assessed the effect of propranolol on antitumor immune response in the MT/Ret mouse model of melanoma. Propranolol treatment delayed primary tumor growth and metastases development in MT/Ret mice. Consistent with our previous observations in human melanoma xenografts, propranolol induces a decrease in cell proliferation and vessel density in the primary tumors and in metastases. In this immunocompetent model, propranolol significantly reduced the infiltration of myeloid cells, particularly neutrophils, in the primary tumor. Inversely, cytotoxic tumor infiltrating lymphocytes were more frequent in the tumor stroma of treated mice. In a consistent manner, we observed the same shift in the proportions of infiltrating leukocytes in the metastases of treated mice. Our results suggest that propranolol, by decreasing the infiltration of immunosuppressive myeloid cells in the tumor microenvironment, restores a better control of the tumor by cytotoxic cells.

## INTRODUCTION

Advances in immunotherapy and chemotherapy improved the handling of metastatic melanoma but the restricted number of responders and the rapid development of resistance limit the scope of these approaches [2–4]. Recent retrospective studies showed a better outcome in melanoma patients under beta-blockers, but these results are still debated [5–8]. Beta-blockers are antagonists for norepinephrine signaling through beta-adrenoceptors. In normal tissue, the stress hormone norepinephrine regulates apoptosis, cell proliferation and angiogenesis [9]. In addition, an increasing number of studies suggest the involvement of norepinephrine signaling in cancer [10–14]. We recently decided to investigate the effect of propranolol, a non-selective beta-blocker, on cancer and particularly on melanoma. Propranolol was discovered in 1960 by James W. Black who received the Nobel Prize in 1988 for this discovery. Now in the public domain, propranolol is a drug of choice for its proven security profile and its low cost which guarantees access to the treatment by the greatest number of people. Several studies reported the role of beta-2 and beta-3 adrenoceptors in various cancers including melanoma [11, 14–17]. Our previous investigations showed a higher efficacy of propranolol over the selective beta-1 adrenoceptor blocker metoprolol in inducing melanoma cell death *in vitro* [18]. The non-selective blocking of beta-adrenoceptors by propranolol may be responsible for its effect on melanoma inhibition. In a xenograft model of human melanoma, our group reported that a treatment with propranolol significantly reduces tumor growth *in vivo* by reducing tumor cell proliferation and intra-tumor vessel density while favoring melanoma cell death [18]. We cannot exclude that the drug also improves the tumor specific immune response. Indeed, norepinephrine induces the release of chemoattractant for myeloid cells and enhances the accumulation of immunosuppressive cells [19–22]. Norepinephrine also induces a myeloid derived suppressor cell (MDSC) phenotype in polymorphonuclear neutrophils (PMNs) while it orientates macrophage polarization to a M2 type [23–25]. In macrophages, norepinephrine inhibits phagocytosis, which limits the immune system activation [26, 27]. In addition, norepinephrine impairs the cytotoxicity of natural killer (NK) cells [28–30], the expansion of memory CD8^+^ T-cells, and promotes a T-Helper 2 lymphocytic response [31]. These observations suggest that norepinephrine may not only promote the survival and proliferation of melanoma cells and the angiogenesis, but also interferes with tumor immune surveillance.

In this context, we investigated the effect of propranolol on anti-tumor immunity in a spontaneous murine model of melanoma. MT/ret mice express constitutively the proto-oncogene c-ret under control of the metallothionein promoter [32]. In this model, a primary tumor develops in the ocular region. Our group described that primary tumor cells disseminate early, remain dormant for several weeks [33], but finally escape the immune surveillance [34, 35] and give rise to macroscopic cutaneous and distant metastases [36]. MDSCs, one of the most abundant hematopoietic population within the primary tumor, and M2-type macrophages support tumor progression via immunosuppressive properties and play a key role in tumor cell dissemination [37, 38]. In contrast with our previous xenograft models, the MT/ret mouse is fully immunocompetent and allows to investigate the effect of propranolol on the immune response.

In the present study, we show that the myeloid infiltration is significantly reduced within the primary tumor and that NK and T-cells appear more cytotoxic under propranolol treatment.

## RESULTS

### A daily propranolol treatment delays primary tumor appearance in MT/ret mice

**Figure 1:**
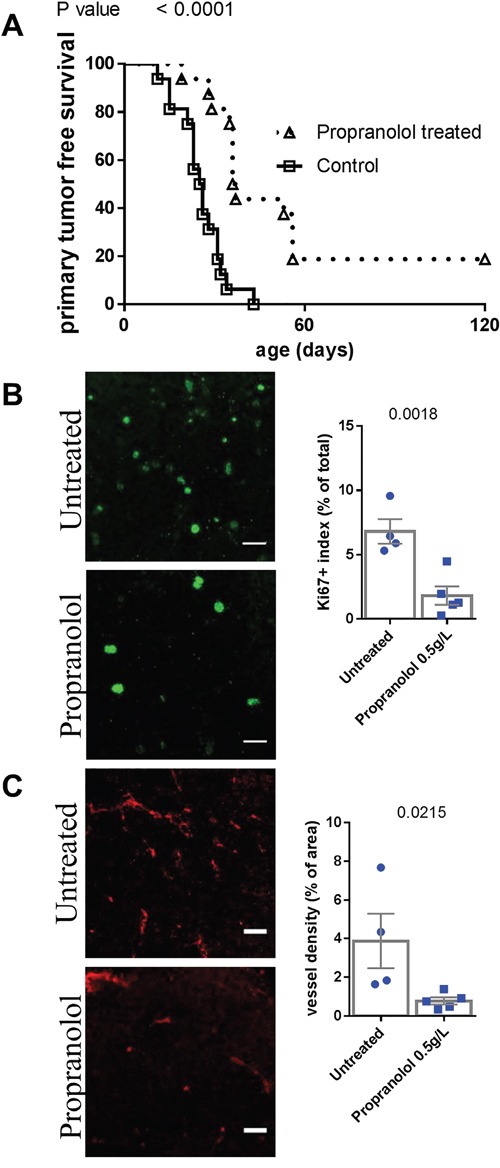
Comparison of tumor development in untreated control and propranolol treated MT/ret mice **A.** Kaplan Meyer survival curves comparing primary tumor development in control (open squares) and propranolol treated (open triangles) animals. **B.** Illustration and quantification of the staining of proliferation marker Ki67 and **C.** the vessel marker CD34 in primary tumors of untreated (top photographs) and propranolol treated (bottom photographs) mice. Mean ± SEM. Scale bar 20 μm.

We assessed the effect of propranolol on the onset of the primary tumor and metastases of melanoma. Propranolol significantly delayed the occurrence of the primary tumor (n=16 mice per group, Figure 1A, Table 1). Three out of sixteen propranolol treated mice never developed a primary tumor, while all control mice did. In accordance with our observations in human melanoma xenografts [18], the treatment is associated with a decreased tumor cell proliferation index as assessed by Ki67 staining (Figure 1B). We observed a reduced intra-tumor vessel density assessed by CD34 staining (Figure 1C).

**Table 1:** Mantel-Cox analysis of primary tumor free, metastasis free and progression free survival

Observed variable	Hazard Ratio	Confidence Interval	P value
Primary tumor free survival	3.8	3.4 to 18.5	<0.0001
Metastasis free survival	2.5	1.38 to 6.6	0.0086
Progression free survival	2.1	1.1 to 5.1	0.0377

### A daily propranolol treatment also delays the appearance of cutaneous metastases in MT/ret mice and prolongs the progression free survival

**Figure 2:**
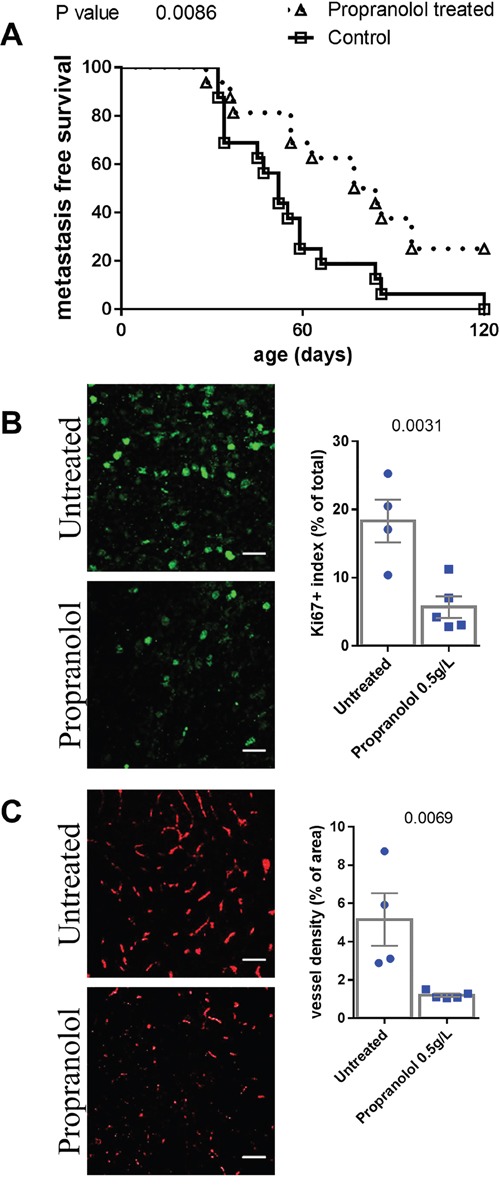
Comparison of metastasis of untreated control and propranolol treated MT/ret mice **A.** Kaplan Meyer survival curves comparing the appearance of metastasis in control (open squares) and treated animals (open triangles). **B.** Illustration and quantification of the staining of proliferation marker Ki67 and **C.** the vessel marker CD34 in primary tumors of untreated (top photographs) and propranolol treated (bottom photographs) mice. Mean ± SEM. Scale bar 20 μm.

Propranolol treatment significantly delayed the occurrence of cutaneous metastases (Figure 2A), Table 1. Besides the three treated MT/ret mice which neither had primary nor distant tumors, one primary tumor bearing mouse from the propranolol-treated group did not develop metastases. In addition, metastases of propranolol treated mice have a lower proliferation index and a reduced vessel density than controls (Figure 2B and 2C).

**Figure 3:**
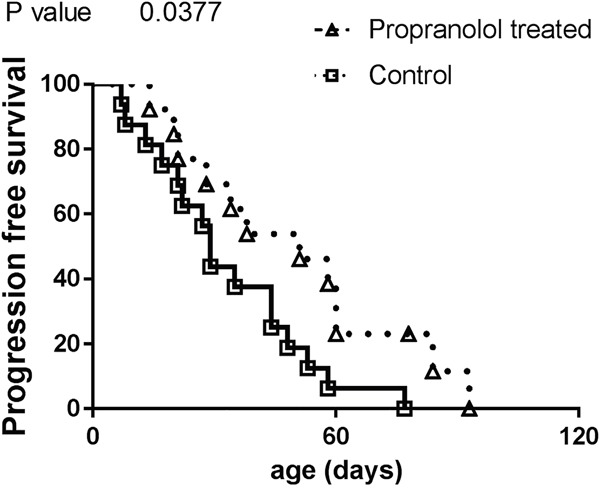
Comparison of progression free survival in control and propranolol treated MT/Ret mice with prior primary tumor Kaplan Meyer survival curves comparing the delay between primary tumor and first metastasis appearance (i.e. progression free survival) in control (open squares) and treated animals (open triangles).

Compared to controls, animals that received propranolol have a longer survival without obvious signs of disease progression (estimated from the detection of primary tumor to the appearance of the first metastasis, propranolol treated mice with no tumor were censored, Figure 3, Table 1).

Altogether, these results strengthen our previous observations showing that propranolol slows down the development of melanoma primary tumor and metastases by affecting proliferation and tumor perfusion.

### Propranolol reduces the myeloid infiltrate in primary tumors

**Figure 4:**
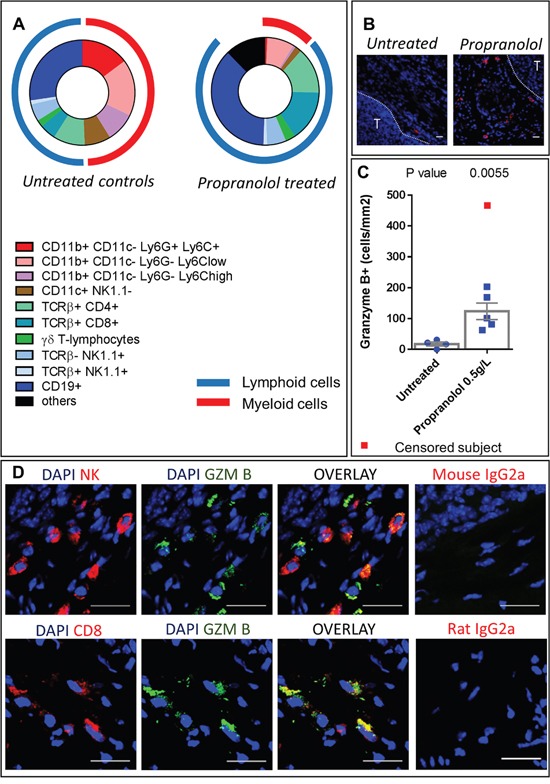
Immune populations infiltrating primary tumors **A.** Diagram showing the distribution of immune populations in the primary tumor of untreated (left) and propranolol treated (right) MT/Ret mice. **B.** Photomicrographs of granzyme B expressing cells in tumor stroma (T for tumor). **C.** Quantification of granzyme B expressing cells in tumors of untreated controls (blue dots) and propranolol treated (blue squares) mice. Red square represents a subject censored because of a high number of granzyme B positive cells in order to not artificially increase the effect size. **D.** Photomicrographs showing the co-expression of NK cell marker (PK136) and granzyme B (top panels) and the co-expression of CD8 T-cell marker and granzyme B (bottom panels) in the tumor stroma of a propranolol treated MT/Ret mouse. Scale bars 20μm. Mean±SEM.

Primary tumor samples were dissociated and we used surface staining of immune cell populations to observe their distribution by flow cytometry. The total number of hematopoietic (CD45+) cells infiltrating the tumor was not significantly different between control and propranolol-treated groups (Table 2). Among hematopoietic cells, the myeloid derived population (CD11b^+^) infiltrating the tumor was significantly reduced from 49% of total hematopoietic cells in control to 11% in propranolol treated animals (raw values are given in Table 2). Furthermore, the treatment significantly reduced different myeloid subsets infiltrating the primary tumor. Among those, the infiltration of PMNs (CD11c^−^Ly6C^+^Ly6G^+^) was significantly inhibited in propranolol treated animals (15% in control vs 0.6% in propranolol group, Figure 4A, Table 2). The treatment also affected macrophages (CD11c^−^Ly6C^lo^Ly6G^−^) which were less represented in the tumors of propranolol treated mice (18% in control vs 8% in propranolol group, Figure 4A Table 2). In the same manner, inflammatory monocytes (CD11c^−^Ly6C^hi^Ly6G^−^) were decreased from 9% in control to 0.8% under propranolol (Figure 4A), Table 2. Finally, we observed a diminution of dendritic cells (CD11c^+^) in primary tumors under propranolol (8% in control versus 2% in propranolol group, Figure 4A, Table 2).

**Table 2:** Quantification of immune cell populations infiltrating the primary tumor

Cell populations	Untreated controls *n=10*	Propranolol treated *n=6*	P-value
CD11b+ ^1^	49.4 ± 8.4	11.2 ± 0.7	0.0001
CD11b+CD11c-Ly6G+Ly6C+ ^1^	15.1 ± 5.1	0.62 ± 0.1	0.0037
CD11b+CD11c-Ly6G-Ly6Clow ^1^	17.7 ± 4.4	8.1 ± 0.4	0.0207
CD11b+CD11c-Ly6G-Ly6Chigh ^1^	9.3 ± 2.1	0.78 ± 0.05	0.0001
Cd11c+ ^1^	8.2 ± 2.0	2.0 ± 0.1	0.0082
CD4+ TCRβ+ ^1^	9.9 ± 1.9	13.9 ± 1.4	ns (0.056)
CD8+ TCRβ+^1^	5.1 ± 0.8	15.3 ± 1.9	0.0001
TCRγδ+^1^	2.2 ± 0.4	3.0 ± 0.2	ns (0.063)
TCRbeta-NK1.1+ ^1^	5.4 ± 1.2	5.8 ± 0.9	ns (0.713)
TCRbeta+NK1.1+ ^1^	1.3 ± 0.35	1.1 ± 0.3	ns (0.813)
Cd19+ ^1^	28.0 ± 3.0	37.1 ± 4.0	0.0215
Cd45+ ^2^	11.3 ± 4.45	12.7 ± 6.65	ns (0.906)

1 Result as a fraction of total hematopoietic cells.

2 Result as a fraction of total dissociated cells.

ns: Not Significant.

These results show that propranolol reduces infiltrating myeloid cells and suggest that it could limit their most harmful counterpart, MDSCs.

### Propranolol promotes B-lymphocytes and granzyme B expressing lymphoid cells in primary tumors

In the lymphoid compartment, we observed a significant increase in B-cells (CD19+) infiltration in primary tumors of propranolol treated MT/Ret mice (28 ± 3 %of CD45+ cells in control vs 37.1 ± 4 in propranolol group, Figure 4A, Table 2). Propranolol significantly increased the infiltration of CD8+ T-cells (5.1 ± 0.8 in control vs 15.3 ± 1.9 in propranolol treated group, Figure 4A, Table 2). In order to investigate in situ the effect of propranolol on anti-tumor cytotoxicity, we assessed the presence of granzyme B by immunohistochemistry. Granzyme B is an apoptosis inducer mainly synthetized by activated cytotoxic T-cells and NK cells. Interestingly, primary tumors from control mice contained very few granzyme B positive cells (17.4 +/− 6.5 cells per mm2, n=4, Figure 4B and 4C). In contrast, these cells were significantly more frequent in all the primary tumors from propranolol treated mice (123.5 +/− 26. 8, n=6, Figure 4B and 4C). Using double labeling of granzyme B / CD8 or granzyme B / NK(PK136), we show that both CD8^+^ T cells and PK136^+^ NK cells express granzyme B (Figure 4D).

Collectively, our data support that propranolol increases B-lymphocytes and CD8^+^ T-cells infiltration of the primary tumor. In particular, propranolol enhanced the fraction of actively cytotoxic CD8^+^ T lymphocytes and NK cells.

### Propranolol treatment decreases PMNs and increases NK and CD8+ T- cytotoxic cells in metastases of MT/ret melanoma

**Figure 5:**
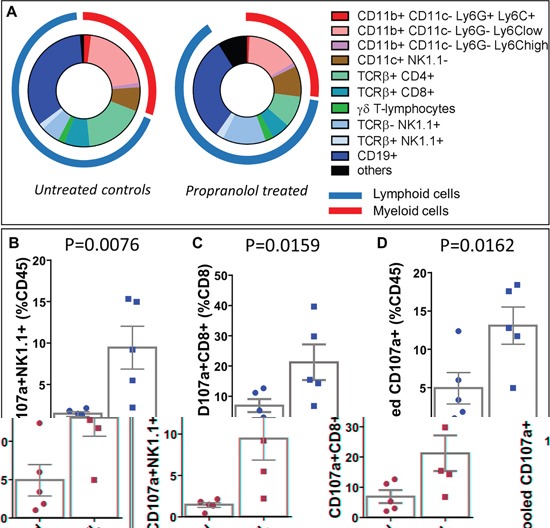
Flow cytometry analyses of immune populations infiltrating metastases and quantification of degranulating cytotoxic cells **A.** Diagram showing the distribution of myeloid and lymphoid cell populations in metastases of untreated control (left panel) and propranolol treated (right panel) MT/Ret mice. **B, C, D.** Flow cytometry quantification of the number of CD107a^+^ degranulating NK cells (B), CD8 T cells (C), or the pooled cytotoxic NK, NK T and CD8^+^ T cells (D) in the metastases of untreated control (blue dots, left bars) and treated (blue squares right bars) MT/Ret mice. Mean ± SEM.

Using flow cytometry analyses of dissociated cells from metastases, we observed a smaller infiltration by hematopoietic cells than in primary tumors in both control and propranolol-treated group. In particular, neutrophilic infiltrate in the metastases derived from untreated mice was moderate in comparison with primary tumors (2% of the total CD45^+^ infiltrate in metastases versus 15% in primary tumors, raw values in Table 2 and Table 3). Nevertheless, the number of PMNs infiltrating the metastases is significantly reduced by propranolol (2% in control vs 0.6% in propranolol group, Figure 5A, Table 3). Propranolol treatment also modulated the lymphocytic infiltrate in the metastases. The number of CD4^+^ T-cells was decreased from 17% in control to 9% in propranolol treated mice (Figure 5A), Table 3. Interestingly, the number of NK cells infiltrating the metastases significantly increased between control animals (5%) and propranolol treated group (13%, Figure 5A, Table 3). The number of CD8^+^ T-cells and CD19^+^ B-cells infiltrating metastases did not change significantly under propranolol treatment (Figure 5A), Table 3. The cytotoxic activity of NK and T-cells was assessed by staining the degranulation marker CD107a in flow cytometry experiments. We observed that propranolol increased the proportion of degranulating NK cells and CD8^+^ T cells in metastases (Figure 5B and 5C). Overall, propranolol increased the fraction of active cytotoxic cells in metastases as shown by pooling CD107a^+^ NK, NK T and CD8^+^ T-cells (Figure 5D).

**Table 3:** Quantification of immune cell populations infiltrating metastases

Cell populations	Untreated controls *n=10*	Propranolol treated *n=13*	P-value
CD11b+ ^1^	28.2 ± 6.2	19.4 ± 2.8	0.1049
CD11b+CD11c-Ly6G+Ly6C+ ^1^	2.0 ± 0.7	0.71 ± 0.1	0.023
CD11b+CD11c-Ly6G-Ly6Clow ^1^	20.6 ± 4.6	15.8 ± 2.9	ns (0.343)
CD11b+CD11c-Ly6G-Ly6Chigh ^1^	1.3 ± 0.4	1.2 ± 0.3	ns (0.205)
Cd11c+ ^1^	7.0 ± 1.9	9.3 ± 1.5	ns (0.390)
CD4+ TCRβ+ ^1^	17.5 ± 2.6	9.4 ± 1.7	0.015
CD8+ TCRβ+^1^	7.3 ± 1.6	5.6 ± 0.9	ns (0.256)
TCRγδ+^1^	2.0 ± 0.5	2.4 ± 0.3	ns (0.242)
TCRbeta-NK1.1+ ^1^	5.4 ± 0.9	13.0 ± 1.6	0.0004
TCRbeta+NK1.1+ ^1^	1.7 ± 0.5	2.3 ± 0.6	ns (0.221)
Cd19+ ^1^	34.0 ± 3.8	31.4 ± 4.8	ns (0.339)
Cd45+ ^2^	3.7 ± 0.7	4.1 ± 0.9	ns (0.390)

1 Result as a fraction of total hematopoietic cells.

2 Result as a fraction of total dissociated cells.

ns: Not Significant.

Altogether, our results suggest that a daily propranolol treatment induces a favorable shift in anti-tumor immunity in metastases by decreasing PMNs infiltration while stimulating the activity of cytotoxic cells.

## DISCUSSION

In the present study we investigated the regulation of anti-tumor immunity by propranolol in melanoma. Propranolol, a non-selective antagonist of beta-adrenoceptors, is suggested to reduce disease progression in different cancer types [6, 7, 39–43]. Because of its dramatic efficacy in the involution of infantile hemangioma [44], it has been proposed that propranolol affects tumor angiogenesis. Recently, we reported in a model of human melanoma xenograft that a daily treatment with propranolol not only reduces tumor vessel density, but also decreases melanoma cells survival and proliferation *in vivo* [18]. In the present study, we highlight the potential of propranolol as a multitarget anti-cancer agent that affects MDSC, tumor associated lymphocytotoxicity as well as melanoma cell proliferation and tumor vessel density.

In order to observe the effect of propranolol on an intact immune system, we used the immunocompetent MT/ret model of spontaneous melanoma. Myeloid cells represent half of the total hematopoietic infiltrate in untreated controls compared to less than 15% in propranolol treated animals. The role of myeloid cells in tumor angiogenesis and cancer progression is widely reported [45–47]. Among myeloid cells, PMN-MDSCs are known to participate to cancer progression and tumor cells dissemination [38, 48–50]. Indeed, beta-2 integrin expressed by PMNs binds to both melanoma and endothelial cells to facilitate the invasion of blood vessels by melanoma cells [49, 50]. Neutrophils also support tumor angiogenesis [51]. In the MT/Ret model, mesenchymal transition of tumor cells and tumor cell dissemination is driven by PMN-MDSCs [38]. The lower number of PMNs infiltrating the primary tumor and metastases in propranolol-treated mice may participate to the decreased angiogenesis (Figure 1C and Figure 2C) and the delay in disease progression (Figure 1A, Figure 2A and Figure 3). Moreover, higher PMN to lymphocytes ratio correlates with a poor response to immunotherapy targeting cytotoxic T lymphocyte antigen 4 and a poor survival [52]. This link between PMN to lymphocytes ratio and disease outcome is reported in different types of cancer [53, 54].

Like PMNs, tumor-associated macrophages stimulate tumor angiogenesis and invasion [55, 56]. Macrophages are also involved in metastatic melanoma resistance to chemotherapy with BRAF inhibitors [4]. Here we observed a significant decrease in the number of macrophages infiltrating the primary tumor but not metastases.

In the stroma of melanoma tumors, a strong immunosuppression mainly due to MDSCs blocks anti-tumor immunity [47]. MDSCs are described as a heterogeneous population of cells comprising immature myeloid progenitors for PMNs, macrophages and dendritic cells. Norepinephrine, the natural agonist of beta-adrenoceptors blocked by propranolol, skews the differentiation of myeloid cells to produce MDSCs, which induce an inhibition of anti-tumor response [13, 20]. Considering the dramatic reduction of the myeloid population in the primary tumor of propranolol treated animals, we hypothesize that propranolol downregulates the MDSC population. MDSC are able to prevent the binding of the T cell receptor with MHC/peptide complexes, to inhibit the signaling in T-cells and to induce T-cell apoptosis [45–47, 57]. Here, we observed a significant increase of CD8^+^ T-cells with a higher expression of granzyme B in the primary tumor stroma of propranolol-treated mice, suggesting a better activation of these cells. In human melanoma, a higher expression of granzyme B in the stroma of the primary tumor is associated with a better outcome [58] and a better response to immunotherapy [59]. In the same way, we found an increased infiltration of NK cells and a higher cytotoxic activity of NK and T-cells in metastases of propranolol treated animals. Propranolol, while reducing the infiltration of myeloid cells and blocking norepinephrine signaling, may limit the differentiation of MDSCs thus decreasing the inhibition of cytotoxic cells. This hypothesis is supported by a study from Jin and colleagues showing that propranolol blocks the accumulation of MDSCs in a model of psychological stress [24]. Moreover, a high level of MDSCs in the peripheral blood of metastatic melanoma patients is correlated with a poor response to immunotherapy targeting Program Death-1 protein [60]. It is reasonable to consider that the decreased infiltration of myeloid populations including MDSC under propranolol may improve the response to immunotherapy. In MT/Ret mice, the inhibition of myeloid cells and the stimulation of cytotoxic cells driven by propranolol are not sufficient to eradicate metastases but we cannot exclude that the constitutive expression of the oncogene *ret*, a neurotrophin receptor, artificially supports tumor progression and immune evasion. Altogether our observations suggest that propranolol decreases immunosuppression in both primary tumors and metastases of MT/ret mice.

On the other hand, dendritic cells are essential mediators of T-cell priming and cytotoxic activation in many infections and cancer. A number of studies report the attempts to manipulate autologous dendritic cells *in vitro* by stimulating antigen presentation before their perfusion to patients [61–63]. Unfortunately, this strategy of dendritic-cell based vaccines, gave rise to a limited success mainly because it failed to significantly stimulate antigen presentation by endogenous cells [63]. Tumor immunosuppressive microenvironment inhibits dendritic cell functions [64, 65]. Especially, norepinephrine released in the microenvironment alters antigen presentation by dendritic cells, which reduces cytotoxic T-cell priming [66, 67]. Here we observed that propranolol decreases the number of dendritic cells in the primary tumor and improves CD8^+^ T-cell activity. It would be interesting to further investigate how propranolol acts on dendritic cell maturation and their subsequent antigen presentation capability.

In humans, the number of B lymphocytes infiltrating the primary tumor or its stroma correlates with a better overall survival and a lower occurrence of metastases in melanoma patients [68, 69]. Ladányi and colleagues show a strong correlation between a high B lymphocyte count and the presence of a high number of activated T-cells as predictors of favorable disease outcome [68]. Furthermore, they report that B lymphocytes participate to antigen presentation and T-cells activation similarly to dendritic cells. Our study shows that propranolol enhanced the infiltration of B lymphocytes within the primary tumor which may reinforce CD8^+^ T-cell cytotoxicity.

In addition to its effect on immune cells, we observed that propranolol decreases melanoma cell proliferation and vessel density inside the primary tumor in this model, consistent with our previous data [18]. Interestingly, we observed that propranolol also decreases proliferation and vessel density inside metastases. We cannot exclude that the observed delay in primary tumor growth and metastases occurrence is only due to this phenomenon but our observations on immune cell populations suggest that propranolol allows a better immune control of the tumor. Furthermore, the decreased myeloid infiltration of the primary tumor, and in particular PMNs and macrophages, may be responsible for the impairment in tumor angiogenesis [51, 55, 56].

Finally, this study brings new insights in the inhibitory effect of propranolol on melanoma development. Besides the direct effect on intra-tumor proliferation and vascularization, propranolol may significantly decrease the infiltration of immunosuppressive cells involved in melanoma progression and bring new tools to overcome resistance to immunotherapy or chemotherapy.

## MATERIALS AND METHODS

### Animal experiments

All experiments were performed in compliance with French Ministry of Agriculture regulations for animal experimentation (number C-75-510). MT/ret mice on the C57Bl/6 genetic background were housed in a specific-pathogens-free environment (Hôpital Cochin, Paris), received either filtered tap water (n=16) or filtered tap water containing 0.5 gram per liter propranolol (n=16, Sigma Aldrich, Germany) as drinking water. We conducted two round of experiments. The first exploratory experiment contained 7 mice per group and 9 mice per group for the second experiment. In propranolol group, mothers were treated during lactation and the treatment was carried on with the young mice studied after weaning. Twice a week propranolol solution was renewed and the appearance of a primary tumor or a cutaneous metastasis was examined. The monitoring consisted in visual examination and manual palpation to observe any bulging suggesting the development of a primary tumor or a cutaneous metastasis nodule. After sacrifice, we confirmed the diagnostic of a tumor when collecting the nodules surgically. After removal, part of the primary tumors and metastases were processed for flow cytometry analyses, the remaining samples were processed for histology.

### Tumor cell suspensions

Tumor samples were mechanically dissociated and enzymatically digested in 1mg/mL collagenase A and 0, 1mg/mL DNase I (Roche, Germany) for 30 min at 37°C only. Cell suspensions of tumors were filtered on a 70μm cell strainer (Sigma Aldrich, Germany), washed in PBS 1X, 5% fetal calf serum (FCS, Gibco, France), 0.5 mM EDTA and resuspended in RPMI 1640 (Gibco).

### Cell surface staining and flow cytometry

Before cell staining, endogenous Fcγ receptors were blocked by a 15-min incubation with a purified anti-CD16/32 antibody (FcγRII/III block; 2.4G2, BD Pharmingen, CA, USA). Surface immunostaining was performed by incubating cells on ice 30 min with either a myeloid populations specific or a lymphoid populations specific antibody cocktail in a solution containing 5% FCS (Sigma Aldrich) and 0.1% NaN3 (Sigma Aldrich) in PBS (antibodies and controls are described in Supplementary Table S1). Multicolor immunofluorescence data were acquired using a LSR2 cytometer (BD Biosciences, CA, USA) and analyzed using Diva software (BD Biosciences).

### Histology and immunostainings

Part of tumor and metastasis samples were paraformaldehyde fixed overnight and paraffin embedded. 10μm sections of paraffin blocks were cut using a microtome (Leica, Germany) and mounted on slides (Superfrost plus, Menzel Glaser, Germany). For immunostainings, slides were deparaffinized in 4 washes of Ultraclear (VWR, Switzerland) for 5 minutes each followed by 4 washes of 3 minutes each in 100% ethanol. Slides were then rinsed in distilled water. Heat induced epitope retrieval in citrate buffer pH6 was performed before staining with the antibodies listed in Supplementary Table S1.

### Image acquisition and quantification

Image acquisition was done on SP5 confocal microscope (Leica, Germany) using stacks of images through the whole section. The images presented here for Ki67, CD34, CD8, PK136 and granzyme B stainings are the result of the whole stack projection. The number of Ki67 positive cells was assessed using imageJ software (NIH, USA). A square of 163 μm x 163 μm was drawn to isolate the highest proliferative area. The number of Ki67 positive cells was calculated automatically using the particle analysis plugin. The total cell count was measured semi-automatically on the same area from a nuclear staining with DAPI and ImageJ. Vessel density was measured as the CD34 staining area to total tumor area ratio. The density of granzyme B positive cells was measured on the whole stromal portion of the section and reported as cells/mm^2^ using image J.

### Statistical analysis

Kaplan Meyer survival curves were generated from mice monitoring data. Odd ratios and significance were estimated by Log rank (Mantel-Cox) analysis. Flow cytometry analyses were compared using Mann-Whitney ranks comparison for each cell type. Histological quantification comparing cell counts between control and propranolol groups were analyzed with unpaired student t-test. Each data are presented as mean +/− SEM and the exact P-value is reported. All analyses were performed with Prism software (GraphPad, CA, USA).

## SUPPLEMENTARY MATERIALS TABLE


