# Supplementary material for: Propranolol induces a favourable shift of anti-tumor immunity in a murine spontaneous model of melanoma
Source: Oncotarget. 2016 Oct 24;7(47):77825–37. doi: 10.18632/oncotarget.12833 (PMC5363624; doi:10.18632/oncotarget.12833)
Supplement: Supplementary file 1 [file oncotarget-07-77825-s001.pdf]

Propranolol induces a favourable shift of anti-tumor immunity in a murine spontaneous model of melanoma

SUPPLEMENTARY TABLE

Supplementary Table S1: Antibodies used for flow cytometry and immunohistochemistry

Target	*Reference / Clone*	*Brand*	*Dilution*	*Negative control*
Antibodies for FACS analyses				
CD107a-PE-Cy7	560647/ 1D4B	BD Pharmingen	1/100	PE-Cy7 Rat IgG2a
CD11b- APC	17-0112-81/M1/70	Ebiosciences	1/500	APC-Rat IgG2b
CD11c-PE-Cy7	558079/HL3	BD Pharmingen	1/100	PE-Cy7 Hamster IgG1
CD19-FITC	557398/ 1D3	BD Pharmingen	1/100	FITC Rat IgG2a
CD4-PB	558107 / RM4.5	BD Pharmingen	1/100	Pacific Blue Rat IgG2a
CD45.2-APC	558702/ 104	BD Pharmingen	1/100	APC mouse IgG2a
CD45.2-PercpCy5.5	552950 / 104	BD Pharmingen	1/100	PercpCy5.5 mouse IgG2a
CD8-APC-H7	560182/ 53-6,7	BD Pharmingen	1/100	APC-H7 Rat IgG2a
Ly6C-v450	560594/ AL-21	BD Pharmingen	1/100	V450 Rat IgM
Ly6G-FITC	551460/ 1A8	BD Pharmingen	1/100	FITC-Rat IgG2a
NK1.1-PE	553165/ PK136	BD Pharmingen	1/100	PE mouse IgG2a
NK1.1-PerCP-Cy5.5	551114/ PK136	BD Pharmingen	1/100	Percp-Cy5.5 mouse IgG2a
TCRß-AF-700	553172/ H57-597	BD Pharmingen	1/100	AF700 armenian hamster IgG2
TCRgd-PE	553178 / GL3	BD Pharmingen	1/100	PE armenian hamster IgG2
Antibodies for Histology experiments				
CD3	Ab16669/SP7	Abcam	1/100	Without primary and CD34
CD34	Ab81289/EP373Y	Abcam	1/200	Without primary and CD3
NK cell	MA1-70100/PK136	Thermofisher	1/100	Mouse IgG2a
CD8 alpha	14-0808-80/4SM15	Ebiosciences	1/50	Rat IgG2a
Granzyme B	ABIN675354	Antibodies online	1/200	Without primary
Ki67	652402/16A8	Biolegend	1/100	Rat IgG2a
Mouse IgG2a isotype	MAB0031	RnD systems	1/100	Without primary
Rat IgG2a isotype	559073	BD Pharmingen	1/50 - 1/100	Without primary
